# Digital clock drawing test reflects visuospatial ability of older drivers

**DOI:** 10.3389/fpsyg.2024.1332118

**Published:** 2024-02-26

**Authors:** Satsuki Yamauchi, Naoko Kawano, Kan Shimazaki, Hiroko Shinkai, Masae Kojima, Kazumitsu Shinohara, Hirofumi Aoki

**Affiliations:** ^1^Institutes of Innovation for Future Society, Nagoya University, Nagoya, Aichi, Japan; ^2^Graduate School of Sustainable System Sciences, Osaka Metropolitan University, Osaka, Japan; ^3^Faculty of Biology-Oriented Science and Technology, Kindai University, Wakayama, Japan; ^4^Graduate School of Human Sciences, Osaka University, Osaka, Japan

**Keywords:** cognitive function, touch screen-based dCDT, on-road driving assessment, aged, driving fitness, visuospatial ability

## Abstract

**Objectives:**

To keep older drivers safe, it is necessary to assess their fitness to drive. We developed a touch screen-based digital Clock Drawing Test (dCDT) and examined the relationship between the dCDT scores and on-road driving performance of older drivers in a community-setting.

**Methods:**

One hundred and forty-one community-dwelling older drivers (range; 64–88 years old) who participated in this study were included in the analysis. Participants completed the dCDT, the Mini-Mental State Examination-Japanese (MMSE-J), and an on-road driving assessment. We examined the relationship between dCDT scores using the method by Rouleau et al. (maximum 10 points) and the on-road driving performance based on a driving assessment system originally developed by Nagoya University.

**Results:**

Multiple regression analyses showed that errors in the driving test were associated with dCDT score for the items “confirmation,” “turning left” and “maintains driving lane position”.

**Discussion:**

This study confirmed the relationship between the dCDT score and driving errors, such as confirmation, turning left and maintaining driving lane position. The increase in these errors indicates a decline in visuospatial ability while driving. The dCDT score may reflect older drivers’ visuospatial abilities while driving.

## 1 Introduction

As the population ages, the number of older drivers is also increasing. Consequently, the number of accidents involving older drivers is also on the rise (Ichikawa et al., 2015). Driving requires abilities such as perception, physical functions, and cognitive functions, all of which decline with age (Anstey et al., 2005). In order to ensure that an older adult can drive safely, it is important to understand their driving fitness. There is thus a need for an efficient test to measure the driving fitness of the increasing number of older drivers.

The Clock Drawing Test (CDT) has been recommended as a screening test for driving fitness of older drivers (Wang et al., 2010). The CDT is a classic neuropsychological test and is used as a screening test to assess cognitive function (Agrell and Dehljn, 1998). There are reports of an association between driving ability and CDT scores in older drivers (Freund et al., 2005, 2008). On the other hand, some reports indicate that CDT has limited utility in predicting driving ability in older drivers (Diegelman et al., 2004; Manning et al., 2014). Thus, there is no consensus on the relationship between driving fitness and high CDT scores among older drivers. The CDT is used for screening for cognitive impairment and dementia, but was originally used to assess visuo-constructive abilities and to measure of spatial dysfunction and neglect (Agrell and Dehljn, 1998). Therefore, one likely explanation for these discrepant results may be that the CDT was originally used to assess visuo-constructive abilities. Previous reports have investigated the relationship between CDT scores and whether older drivers are safe to drive (Freund et al., 2005), or whether CDT performance can predict the occurrence of accidents (Diegelman et al., 2004). Driving is a very complicated task that requires multiple cognitive functions, including attention, visual perception, and visuospatial abilities (Reger et al., 2004), to deal with the various situations that occur during driving. Tinella et al. (2022) explained that both egocentric (self-to-object) and allocentric (object-to-object) spatial representations are assumed to be crucial for driving skills. However, we have not found any reports that have investigated the types of driving situations that CDT performance reflects. The CDT may reflect the visuospatial abilities, one of the various abilities required for driving, rather than the driving fitness of older drivers. Clarifying what driving conditions the CDT reflects may help establish a validated desk-based screening test to measure driving fitness of older drivers. With the current increase in the number of older drivers, it is important to identify appropriate driving fitness tests and reduce the cost of assessing older drivers.

The CDT, using paper-and-pencil, is easily and quickly tested. However, several different scoring methods have been developed for the CDT (Spenciere et al., 2017), and scoring methods are not unified (Matsuoka et al., 2011). In addition, adequate training and scoring time for the scorers is required (Carr et al., 2011). Recently, a digital version of the CDT (dCDT) has been developed as an alternative to the classic pencil-and-paper CDT, and its usefulness as a new neuropsychological test has been reported (Souillard-Mandar et al., 2016; Müller et al., 2019; Dion et al., 2020; Yuan et al., 2021). If a system that can automatically scoring dCDT can be constructed, the burden related to scoring can be eliminated. Furthermore, compared to CDT using paper-and-pencil, dCDT have various variables such as total time, speed, writing pressure (Müller et al., 2019), and clock face area (Dion et al., 2020) that can detect slight cognitive decline not detected by the CDT.

Therefore, we developed a dCDT using a touch screen monitor and hypothesized that a dCDT performance would be related to visuospatial abilities required for driving. The purpose of this study was to investigate the relationship between dCDT performance and on-road driving assessments that reflect the visuospatial abilities in older drivers.

## 2 Methods

### 2.1 Participants

Study participants were recruited from the Data Repository for Human Life-Driving Anatomy (“Dahlia”), a longitudinal analysis that collects community-dwelling data at the baseline and annual follow-up of a prospective cohort study ongoing since 2014 (Yamagishi et al., 2019). Dahlia participants were recruited through a snowball sampling design to effectively recruit older drivers who drive regularly and ensure their participation in the measurement (preventing cancelations). They were measured various human characteristic data related to driving. Participants diagnosed with dementia were excluded from this study. The data for this study used 142 individuals (range; 64–88 years, male; *N* = 79, female *N* = 63) from the Dahlia data measured in 2021, who participated in both the cognitive test and the on-road driving assessment. Informed consent was obtained from all participants. The study received ethical approval from the Institute of Innovation for Future Society at Nagoya University.

### 2.2 Touch screen-based dCDT

**Figure 1:**
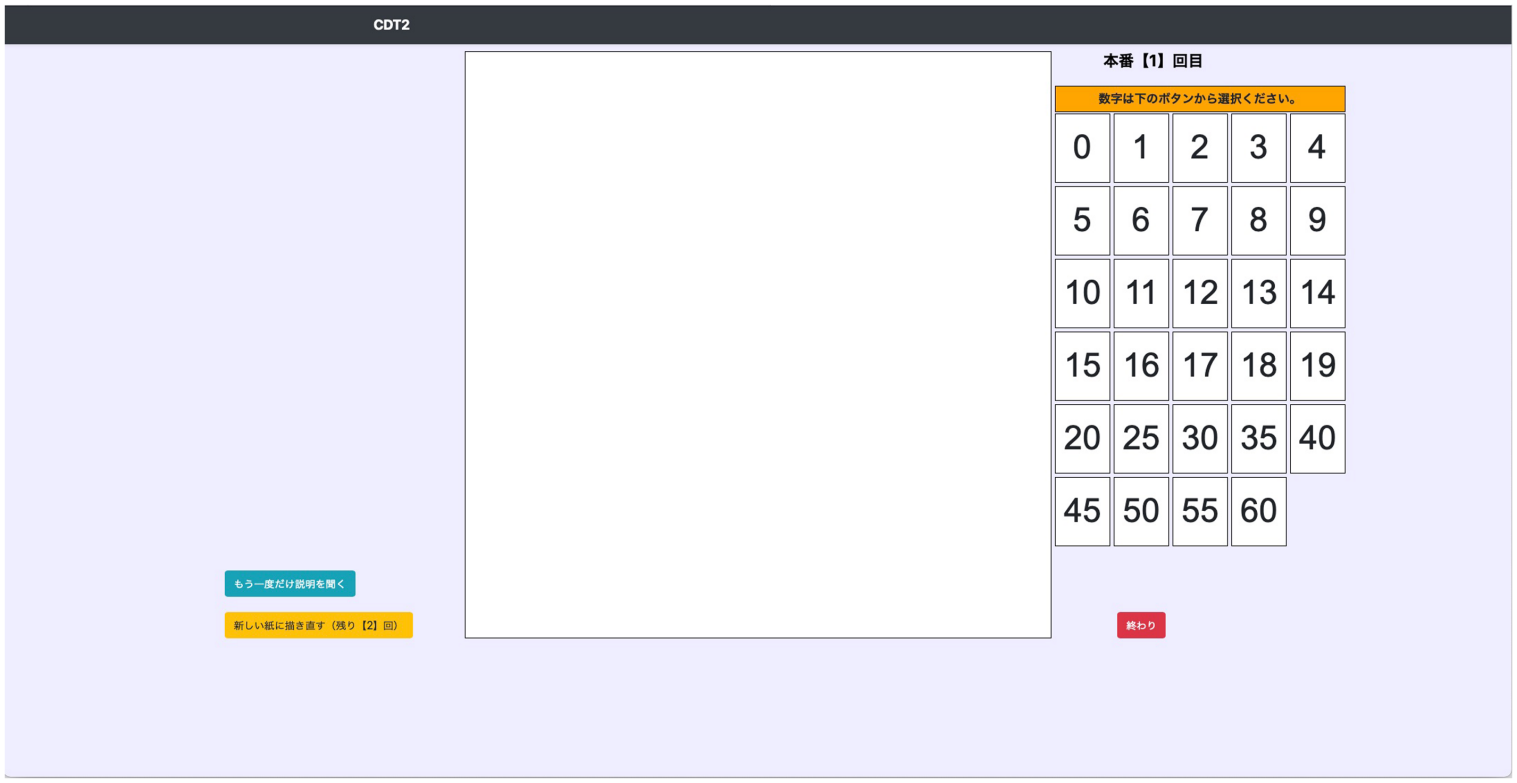
Test screen of the touch screen-based digital clock drawing test. The blank drawing area is 21 cm × 21 cm, and numbers from 1 to 60 are provided on the right side of it in order to easily distinguish the placed numbers. Participants drew the clock face and hands with their fingers, and the numbers were selected from the numbers provided on the right side and dragged and dropped.

We developed the dCDT using a 21.5-inch touch screen (ThinkCentre M820z, Lenovo). The blank drawing area is 21 cm × 21 cm, and numbers from 1 to 60 are provided on the right side of it in order to easily distinguish the placed numbers (Figure 1). First, the participants drew their names and dragged and dropped the numbers of their birthdays on the screen to familiarize themselves with the touch screen operation with their fingers. This phase was not scored in the study. Next, participants were told that they were now going to draw a clock and that the clock numbers were to be selected from the numbers provided on the right side and dragged and dropped. Then, they were given verbal instructions: “Please draw a picture of a clock at ten past eleven with all the numbers inside of the circle.” There was no time limit for drawing, and participants could revise their drawings up to three times when they felt it was necessary. The dCDT was scored using the method of Rouleau et al. (1992), and was performed independently by two researchers, one who was present during the dCDT test and one who was not involved in the dCDT test and did not know the participants’ characteristics. The 10-point scale for the Rouleau CDT is designed to independently assess the accuracy of the clock face representation (maximum 2 points), the arrangement of the numbers (maximum 4 points) and the position of the hands (maximum 4 points) (Appendix 1). If two investigators’ scores differed, they conferred to determine the score. Weighted Kappa statistics were calculated to assess the inter-rater reliability of the dCDT score between the two raters. The inter-rater reliability of dCDT scores was 0.94 (95% *CI* = [0.94, 0.94]), indicating substantial inter-rater agreement.

### 2.3 MMSE-J

The Mini-Mental State Examination-Japanese (MMSE-J) was used to assess the cognitive status of the participants. The MMSE is a widely used screening test for dementia, and its Japanese version is the MMSE-J. The cut-off score for suspected dementia on the MMSE-J is 23 (Sugishita et al., 2018). In this study, therefore, data from one participant with an MMSE-J score of 23 or less were excluded.

### 2.4 On-road driving assessment

Participants completed an on-road driving test approximately 7.2 km in distance, taking approximately 30 min, starting and ending at Nagoya University. In Japan, vehicles drive on the left side of the road. The route included four-lane roads, two-lane roads, roads without centerlines, curves, right and left turns, temporary stops, and traffic signals, and all participants drove the same route. In this study, a single driving instructor with 16 years of instructor experience performed all driving evaluations. She is a nationally certified driving instructor. The driving instructor sat in the front passenger seat and provided directions for the driving route and maintained traffic safety as needed. The cognitive status of drivers was concealed from the driving instructor.

The driving assessment system was originally developed by Nagoya University, and assessments were conducted as follows. The driving instructor sat in the front passenger seat and assessed driving behavior while driving using a tablet connected to the Internet. The tablet screen had buttons for assessment items created based on the criteria for the driver’s license examinations, and if an error was observed while driving on the road, the assessor pressed the appropriate button. The date and time, location information, and which button was pressed were recorded once per second and stored in the tablet. At the on-road driving test, the assessment data were transferred to the cloud and stored when the end button was pressed. Due to their widely varying characteristics, the on-road assessment data in this study were categorized and analyzed according to common items. A list of the categorized error items is shown in Table 1. The sum of error items for each category was used for analysis.

**Table 1:** Error items classified by common items in the on-road assessment.

Error item	Description of error item
Overall	Total number of failures during on-road driving.
Operation	Inappropriate gas pedal, brake pedal, or steering wheel operation. For example, sudden acceleration/braking or steering wheel wobble.
Confirmation	Lack of safety confirmation behavior at intersections and lane changes.
Stop intersection	Not stopping at stop lines or before intersections.
Turning left	Total number of failures that occur when turning left. For example, lack of safety confirmation behavior when turning left or failure to pull over to the left side sufficiently.
Turning right	Total number of failures that occur when turning right. For example, lack of safety confirmation behavior when turning right or failure to pull over to the right side sufficiently.
Signals in time	Signaling when changing course is not appropriate.
Traffic light	Ignoring red and yellow lights.
Risk prediction	Failure to gather information and make predictions for safe and smooth driving. For example, inappropriate gap selection, lack of scanning techniques and lack of blind spot checking.
Maintaining distance between vehicles	Failure to maintain appropriate distance between vehicles. For example, stopping too closely behind cars or following other cars too closely.
Maintaining driving lane position	Wobbling to the left or right in the driving lane or driving too close to (or over) the centerline or left line.
Maintaining appropriate speed	Driving above the legal or regulated speed limit.

### 2.5 Statistical analysis

This analysis is based on a portion of a longitudinal study, so the sample size was not calculated *a priori*. According to the *post hoc* power analysis (using G*power 3.1, significance level *α* = 0.05, effect size in multivariable linear regression analysis *R^2^* = 0.07, 0.09, 0.17 (for confirmation, turning left, maintaining driving lane position, respectively), number of data *N* = 141), power (1-*β*) = 0.65, 0.79, 0.99 (for confirmation, turning left, maintaining driving lane position, respectively).

Spearman’s rank correlation coefficient was calculated to examine relationships between the dCDT score and driving evaluation variables. Multivariable regression analysis was used to examine the effect of the dCDT score on on-road driving assessment items. Data were analyzed using JMP® Pro 16.1.0 (SAS Institute Inc.).

## 3 Results

Participant characteristics are summarized in Table 2. The total number of participants analyzed was 141, excluding one person whose MMSE score was 23. Many participants drove cars on a daily basis (everyday; 57%, once every 2–3 days; 31%). The mean MMSE-J score was 28.4 (range; 24–30). The mean dCDT score was 7.1 (range; 1–10).

**Table 2:** Participant characteristics.

	Overall	Male	Female
Number of participants, *n* (%)	141	78 (55)	63 (45)
Age (years), mean (*SD*)	75.0 (5.9)	76.2 (5.6)	73.5 (6.0)
Education (years), mean (*SD*)	13.0 (2.2)	13.2 (2.5)	12.7 (1.7)
Driving frequency, *n* (%)			
Less than once per month	0 (0)	0 (0)	0 (0)
Once every 2–3 weeks	4 (3)	3 (2)	1 (1)
Once a week	13 (9)	7 (5)	6 (4)
Once every 2–3 days	44 (31)	23 (16)	21 (15)
Every day	80 (57)	45 (32)	35 (25)
Driving distance per week, *n* (%)			
0 km	0 (0)	0 (0)	0 (0)
1–10 km	32 (23)	13 (9)	19 (13)
11–50 km	59 (42)	27 (19)	32 (23)
51–100 km	35 (25)	25 (18)	10 (7)
101–300 km	12 (9)	11 (8)	1 (1)
301 km ≤	3 (2)	2 (1)	1 (1)
MMSE-J score, mean (*SD*)	28.4 (1.6)	28.2 (1.6)	28.7 (1.5)
dCDT score, mean (*SD*)	7.1 (2.1)	6.8 (2.1)	7.3 (2.0)

MMSE-J, Mini-Mental State Examination-Japanese; dCDT, digital Clock Drawing Test; SD, standard deviation.

Spearman’s rank correlation coefficients between the dCDT score and the on-road driving assessment are shown in Table 3. Significant negative correlations were found between the dCDT score and errors in confirmation (*rs (141)* = −0.210, *p*-value = 0.013), turning left (*rs (141)* = −0.184, *p*-value = 0.029), and maintaining driving lane position (*rs (141)* = −0.210, *p*-value = 0.012), although the effect sizes were small. A significant positive correlation was also found between the dCDT scores and the error in maintaining appropriate speed (*rs (141)* = 0.263, *p*-value = 0.002), although the effect size was small. On the other hand, there was no significant correlation between the dCDT score and the overall error in the on-road driving assessment.

**Table 3:** Spearman’s rank correlation coefficient between the dCDT score and error items of the on-road driving assessment.

Error item	*rs*	*p-*value
Overall	−0.028	0.741
Operation	0.137	0.105
Confirmation	−0.210	0.013
Stop intersection	−0.071	0.403
Turning left	−0.184	0.029
Turning right	−0.144	0.090
Signals in time	−0.040	0.642
Traffic light	−0.080	0.346
Risk prediction	0.052	0.537
Maintaining distance between vehicles	0.013	0.882
Maintaining driving lane position	−0.210	0.012
Maintaining appropriate speed	0.263	0.002

To determine the effect of dCDT scores on the error items for which correlations were found, we performed a multivariable linear regression analysis. We entered age, sex, education, estimated driving distance per year (median scale of driving distance per week converted to per year), MMSE-J and dCDT score into this model. These variables are known to be associated with driving performance and cognitive function (Anstey et al., 2005; Freund et al., 2005, 2008; Adrian et al., 2011; Ichikawa et al., 2015). We found that the dCDT score was a significant predictor of errors in confirmation (*β* = −0.229, *p*-value = 0.012), turning left (*β* = −0.241, *p*-value = 0.007), and maintaining driving lane position (*β* = −0.320, *p*-value = 0.000). The proportion of variance explained by age, gender, education, estimated driving distance per year, and MMSE-J, respectively, is as follows: confirmation; *R^2^* = 0.023, turning left; *R^2^* = 0.042, and maintaining driving lane position; *R^2^* = 0.085. In contrast, dCDT score was not a predictor of error in maintaining appropriate speed, but sex was a significant predictor (*β* = 0.191, *p*-value = 0.039), with male drivers making more errors in maintaining appropriate speed (Table 4).

**Table 4:** Liner regression assessing variables association with error item during on-road driving.

	Error item							
	Confirmation		Turn left		Maintains driving lane position		Maintains appropriate speed	
	*R^2^=* 0.069		*R^2^=* 0.094		*R^2^=* 0.174		*R^2^=* 0.085	
	*β*	*p*-value	*β*	*p*-value	*β*	*p*-value	*β*	*p*-value
Predictor variable	[95%*CI*]		[95% *CI*]		[95% *CI*]		[95% *CI*]	
Age (years)	0.115	0.216	0.075	0.411	0.196	0.026	−0.074	0.418
	[−0.09, 0.38]		[−0.12, 0.30]		[0.02, 0.25]		[−0.19 0.08]	
Gender (F)	0.086	0.350	0.196	0.032	0.089	0.303	0.213	0.020
	[−0.73, 2.06]		[0.12, 2.64]		[−0.33, 1.05]		[0.15, 1.73]	
Education (years)	0.136	0.122	0.110	0.202	0.067	0.420	0.008	0.929
	[−0.13, 1.08]		[−0.19, 0.89]		[−0.18, 0.42]		[−0.32, 0.36]	
Estimated driving distance per year (10,000 km)	0.052	0.566	−0.032	0.716	0.111	0.192	0.122	0.173
	[−2.94, 5.37]		[−4.43, 3.05]		[−0.69, 3.41]		[−0.72, 3.98]	
MMSE-J	0.064	0.489	0.027	0.765	−0.077	0.381	−0.155	0.093
	[−0.57, 1.18]		[−0.67, 0.91]		[−0.62, 0.23]		[−0.92, 0.07]	
dCDT score	−0.231	0.011	−0.246	0.006	−0.322	0.000	0.128	0.155
	[−1.54, −0.20]		[−1.44, −0.24]		[−0.96, −0.30]		[−0.10, 0.65]	

F, female; MMSE-J, Mini-Mental State Examination-Japanese; dCDT, digital Clock Drawing Test; *R*^2^, coefficient of determination; *β*, beta coefficient; CI, confidence interval.

## 4 Discussion

This study aimed to examine the relationship between dCDT and driving performance in older drivers. We found a relationship between dCDT score and driving error, such as errors in confirmation, turning left and maintaining driving lane position. These situations require visuospatial cognitive skills while driving, such as intersections and lane changes. Errors in turning left include failing to pull the vehicle sufficiently to the left when making a left turn. In Japan, vehicles drive on the left side of the road, and turning left require more visuospatial ability while driving than turning right. Also, it is known that with decreasing arousal and aging, it becomes more difficult to direct attention to the left side of space than to the right side (Chandrakumar et al., 2019). For these reasons, in this study, only errors in turning left, not turning right, may have correlated with dCDT. It is well known that the CDT covers the assessment of comprehension, planning, visual memory, recalibration of graphic images, visuospatial function and execution, number recognition, abstraction, and concentration, but it was originally used to assess visuospatial ability (Agrell and Dehljn, 1998). Using neuroimaging studies Matsuoka et al. (2011, 2013) reported that the Rouleau CDT score appears mainly to reflect functions of the bilateral posterior temporal lobe and the right parietal lobe, including patients with Alzheimer’s disease (AD). These cortical lobes are responsible for visuospatial ability and semantic memory. Thus, the results of this study support their reports and the dCDT scores may reflect the types of driving situations that require visuospatial cognitive abilities (e.g., confirmation, turning left and maintaining lane position).

In addition to visuospatial abilities, multiple cognitive abilities are essential for safe driving, including complex attention, working memory, executive function, judgment, and insight into one’s own driving abilities (Hill et al., 2019). Previous studies have found no consensus on the relationship between driving fitness and CDT scores in older drivers (Diegelman et al., 2004; Freund et al., 2005, 2008). On the other hand, some previous reports have shown that driving fitness can be predicted by combining multiple off-road tests, including CDT. Carr et al. (2011) reported that a screening battery including the CDT, the Eight-item Informant Interview to Differentiate Aging and Dementia, and time to complete the Snellgrove Maze Test or Trail Making Test (TMT) Part A predicted driving fitness in drivers with dementia in an on-road driving evaluation clinical setting at an academic rehabilitation center. A recent study reported that combining CDT and TMT may increase the predictability of driving cessation (Kokkinakis et al., 2021). Therefore, combining the dCDT, which measures visuospatial ability, with neurophysiological tests that measure other cognitive functions may allow for the screening of driving fitness in older drivers.

The error in maintaining an appropriate speed was correlated with dCDT. However, multivariable regression analysis was used to control for sex and found to be related to sex, but not to dCDT score. The reason for this was that dCDT score was lower for males than for females. In previous studies of non-older drivers (Li et al., 2015; Lyu et al., 2017), male drivers drove at higher speeds than female drivers. The results of this study in older drivers also support these reports.

Besides cognitive function, there are several other factors that affect the driving fitness of older drivers. For example, inattentive tendencies due to attention deficit-hyperactivity disorder (ADHD) negatively affect driving behavior (Barkley and Cox, 2007; Biederman et al., 2007), and these influences cannot be ruled out in this study. Therefore, future studies comparing older drivers with ADHD tendency and those without ADHD tendency may be necessary (Nagano et al., 2023).

This study developed a touch screen-based dCDT, but there are technical limitations. The dCDT can provide more detailed information such as total time, speed, writing pressure, and clock face area, than the traditional paper-and-pencil CDT (Souillard-Mandar et al., 2016; Müller et al., 2019; Dion et al., 2020; Yuan et al., 2021). Dion et al. (2020) reported that participants with mild cognitive impairment took longer to finish drawing the clock and tended to draw a smaller area of the clock face than participants with normal cognitive abilities. In addition, other studies have used machine learning to automatically score dCDT performance, similar to traditional scoring systems (Souillard-Mandar et al., 2016). These reports allow for the elimination of inconsistencies in scoring by scorers and the burden caused by time-consuming scoring. Furthermore, Rentz et al. (2021) reported that dCDT results using a digital pen and special paper with a faint grid pattern were associated with amyloid and tau burden, early biomarkers of AD, in clinically normal older adults. They mention that a more detailed examination of dCDT performance may provide insight into early cognitive decline that occurs before diagnosis. In addition, Müller et al. (2019) showed that a tablet-based dCDT parameter is a suitable screening tool for identifying early cognitive impairment, and its performance is comparable to the Consortium to Establish a Registry for Alzheimer’s Disease test battery, a time-consuming and established neuropsychological method. In the future, we plan to develop a system to automatically calculate dCDT scores. In this study, the dCDT was scored using the method of Rouleau et al. (1992), but further studies adding various detailed information obtained from the dCDT may make it possible to understand not only visuospatial ability but also other cognitive functions necessary for driving, and to identify earlier the decline in driving ability associated with cognitive decline.

A further limitation is that older people are not accustomed to operating touch screens. To solve this problem, this study added trials in which participants drew their own name and dragged and dropped birthday number with their fingers before starting dCDT.

In conclusion, we developed the touch screen-based dCDT and show that the dCDT score may reflect the visuospatial ability of older drivers, thus that it can be used as a driving assessment for older drivers. However, the dCDT does not appear to reflect overall driving fitness. It should be used in combination with other neuropsychological tests to measure overall driving fitness in older drivers.

## Data availability statement

The raw data supporting the conclusions of this article will be made available by the authors, without undue reservation.

## Ethics statement

The studies involving humans were approved by Ethics committee of the Institute of Innovation for Future Society at Nagoya University. The studies were conducted in accordance with the local legislation and institutional requirements. The participants provided their written informed consent to participate in this study.

## Author contributions

SY: Conceptualization, Data curation, Formal analysis, Writing – original draft. NK: Conceptualization, Formal analysis, Writing – original draft, Writing – review & editing. KanS: Conceptualization, Writing – review & editing. HS: Data curation, Writing – review & editing. MK: Data curation, Writing – review & editing. KazS: Funding acquisition, Project administration, Writing – review & editing. HA: Conceptualization, Funding acquisition, Project administration, Writing – review & editing.
