# Supplementary material for: Digital clock drawing test reflects visuospatial ability of older drivers
Source: Front Psychol. 2024 Feb 26;15:1332118. doi: 10.3389/fpsyg.2024.1332118 (PMC10925675; doi:10.3389/fpsyg.2024.1332118)
Supplement: Supplementary file 1 [file Table_1.DOCX]

Supplementary Material

# Supplementary Tables

## Appendix 1. Scale for scoring clock drawings using the 10-point rating scale of Rouleau et al

## Appendix 2. List of names of each error button on the driving assessment tablet

| Error item |  | Names of each error button |  |
| --- | --- | --- | --- |
| Operation |  | Gas pedal operation |  |
|  |  | Brake pedal operation |  |
|  |  | Steering wheel operation |  |
| Confirmation |  | Turn right at a stop intersection | Crosswalk |
|  |  |  | Crossroad |
|  |  |  | Sidewalk |
|  |  |  | Oncoming vehicle |
|  |  | Turn left at a stop intersection | Crosswalk |
|  |  |  | Crossroad |
|  |  |  | Sidewalk |
|  |  |  | Turn without hitting anything |
|  |  | Go straight at a stop intersection | Crosswalk |
|  |  |  | Crossroad |
|  |  |  | Sidewalk |
|  |  |  | Oncoming vehicle |
|  |  | Change lane to the right | Confirmation |
|  |  | Turning right | Crosswalk |
|  |  |  | Crossroad |
|  |  |  | Oncoming vehicle |
|  |  | Change lane to the left | Confirmation |
|  |  | Turning right | Crosswalk |
|  |  |  | Crossroad |
|  |  |  | Turn without hitting anything |
|  |  | Turning right at a traffic light | Crosswalk |
|  |  |  | Check the lead vehicle |
|  |  |  | Oncoming vehicle |
|  |  | Turning left at a traffic light | Crosswalk |
|  |  |  | Turn without hitting anything |
|  |  |  | Check the lead vehicle |
|  |  | Go straight | Check the lead vehicle |
| Stop intersection |  | Turning right at a stop intersection | Stopping at stop lines |
|  |  |  | Gradually drive forward with care |
|  |  | Turning left at a stop intersection | Gradually drive forward with care |
|  |  |  | Stop before the stop line |
|  |  | Go straight at a stop intersection | Stopping at stop lines |
|  |  |  | Gradually drive forward with care |
|  |  | Turning right at a traffic light | Stopping at stop lines |
|  |  | Turning left at a traffic light | Stopping at stop lines |
| Turning left |  | Turning left at a stop intersection | Swing right, then turn left. |
|  |  |  | Crosswalk |
|  |  |  | Crossroad |
|  |  |  | Sidewalk |
|  |  |  | Turn without hitting anything |
|  |  |  | Gradually drive forward with care |
|  |  |  | Pull over to the left and turn |
|  |  |  | Stopping at stop lines |
|  |  |  | Not pulled over to the left |
|  |  | Change to the left lane | Confirmation |
|  |  |  | Signal |
|  |  | Turning left at a traffic light | Swing right, then turn left. |
|  |  |  | Crosswalk |
|  |  |  | Turn without hitting anything |
|  |  |  | Check the lead vehicle |
|  |  |  | Make a big turn |
|  |  |  | Stopping at stop lines |
|  |  |  | Pull over to the left and turn |
| Turning right |  | Turning right at a stop intersection | Through oncoming lane |
|  |  |  | Stopping at stop lines |
|  |  |  | Crosswalk |
|  |  |  | Crossroad |
|  |  |  | Swing left, then turn right. |
|  |  |  | Sidewalk |
|  |  |  | Gradually drive forward with care |
|  |  |  | Oncoming vehicle |
|  |  |  | Pull over to the right and turn |
|  |  | Turning right | Shortcut and turn |
|  |  |  | Crosswalk |
|  |  |  | Crossroad |
|  |  |  | Swing left, then turn right |
|  |  |  | Oncoming vehicle |
|  |  |  | Pull over to the right and turn |
|  |  | Turning right at a traffic light | Shortcut and turn |
|  |  |  | Crosswalk |
|  |  |  | Swing left, then turn right. |
|  |  |  | Check the lead vehicle |
|  |  |  | Oncoming vehicle |
|  |  |  | Stopping at stop lines |
|  |  |  | Pull over to the right and turn |
| Signals in time |  | Change to the left lane |  |
|  |  | Change to the left lane |  |
| Traffic light |  | Yellow light |  |
|  |  | Red light |  |
| Risk prediction |  | Anticipating traffic conditions |  |
|  |  | Appropriate judgment |  |
| Maintaining distance between vehicles |  | Maintaining distance between vehicles |  |
| Maintaining driving lane position |  | Maintaining driving lane position |  |
| Maintaining appropriate speed |  | Maintaining appropriate speed |  |
